# Molecular biomarkers predicting newly detected atrial fibrillation after ischaemic stroke or TIA: A systematic review

**DOI:** 10.1177/23969873221136927

**Published:** 2022-12-06

**Authors:** Kirsty Ward, Andy Vail, Alan Cameron, Mira Katan, Gregory YH Lip, Jesse Dawson, Craig J Smith, Amit K Kishore

**Affiliations:** 1 Manchester Centre for Clinical Neurosciences, Geoffrey Jefferson Brain Research Centre, Manchester Academic Health Science Centre, Salford Care organisation, Northern Care Alliance NHS Foundation Trust, UK; 2 Centre for Biostatistics, University of Manchester, Manchester Academic Health Science Centre, UK; 3 Institute of Cardiovascular and Medical Sciences, University of Glasgow, Glasgow, UK; 4 Stroke Center/Dept. Of Neurology University Hospital and University of Basel, Switzerland; 5 Stroke Center/Dept. Of Neurology University Hospital and University of Zurich, Switzerland; 6 Liverpool Centre for Cardiovascular Science at University of Liverpool, Liverpool John Moores University and Liverpool Heart & Chest Hospital, Liverpool, UK; 7 Department of Clinical Medicine, Aalborg University, Aalborg, Denmark; 8 Division of Cardiovascular Sciences, Faculty of Biology, Medicine and Health, University of Manchester, Manchester, UK

**Keywords:** Ischaemic stroke, transient ischaemic attack, molecular biomarker, atrial fibrillation

## Abstract

**Background:**

Several molecular biomarkers are available that predict newly detected atrial fibrillation (NDAF). We aimed to identify such biomarkers that predict NDAF after an Ischaemic stroke (IS)/Transient Ischaemic Attack (TIA) and evaluate their performance.

**Methods:**

A systematic review was undertaken in accordance with the Preferred Reporting Items for Systematic Reviews and Meta-Analyses (PRISMA) statement. Studies of patients with IS, TIA, or both, who underwent ECG monitoring for ⩾24 h, which reported molecular biomarkers and frequency of NDAF after electronic searches of multiple databases were included.

**Results:**

Twenty-one studies (76% IS, 24% IS and TIA) involving 4640 patients were included. Twelve biomarkers were identified, with cardiac biomarkers evaluated in the majority (75%) of patients. Performance measures were inconsistently reported. Among cohorts selecting high-risk individuals (12 studies), the most studied biomarkers were N-Terminal-Pro Brain Natriuretic Peptide (NT-ProBNP, five studies; C-statistics reported by three studies, 0.69–0.88) and Brain Natriuretic Peptide (BNP, two studies; C-statistics reported in two studies, 0.68–0.77). Among unselected cohorts (nine studies), the most studied biomarker was BNP (six studies; C-statistics reported in five studies, 0.75–0.88). Only BNP was externally validated (two studies) but using different thresholds to categorise risk of NDAF.

**Conclusion:**

Cardiac biomarkers appear to have modest to good discrimination for predicting NDAF, although most analyses were limited by small, heterogeneous study populations. Their clinical utility should be explored further, and this review supports the need to assess the role of molecular biomarkers in large prospective studies with standardised selection criteria, definition of clinically significant NDAF and laboratory assays.

## Introduction

Newly detected atrial fibrillation (NDAF), which most commonly refers to occult or paroxysmal atrial fibrillation (PAF) is a frequent cause of cardioembolism,^1–4^ but is often difficult to detect due to brevity of episodes and its frequently asymptomatic nature.^4^ NDAF after ischaemic stroke (IS)/transient ischaemic attack (TIA) has been variably reported with extended electrocardiography (ECG) monitoring^5^ and treatment with anticoagulation is not recommended for cryptogenic stroke patients unless atrial fibrillation (AF) or another indication for anticoagulation is present.^6,7^ Although extended ECG monitoring is recommended in most societal guidelines for detection of new AF, there is lack of clarity and consensus on duration of ECG monitoring, timing of ECG monitoring after IS/TIA, and patient selection for extended ECG monitoring.^8–10^ Studies of invasive cardiac monitoring with an implanted loop recorder (ILR) following cryptogenic stroke detected PAF in ~20% of cases after 2 years,^11,12^ but routine use has logistical and resource implications.

Molecular biomarkers have been studied in relation to haemostasis, cardiac strain and dilation, and osmoregulation (Brain Natriuretic peptide, BNP; Atrial Natriuretic Peptide, ANP), endothelial damage, thrombogenesis (fibrinogen, d-dimer), and inflammatory processes (Interleukin, IL-8; C-reactive protein, CRP) in AF and cardioembolic strokes, but their clinical utility in stroke patients remains to be defined.^13–15^ If a molecular biomarker could reliably predict NDAF following IS/TIA, it could help to tailor investigation by risk-stratifying people who may benefit from prolonged ECG monitoring as well as potentially identifying people less likely to have NDAF and who can safely have a less intensive approach to investigation. Further, the ease of blood sampling and availability of point-of-care testing for certain molecular biomarkers provide convenience for testing in the acute or outpatient setting.^16^ We therefore performed a systematic review, with the overall aim to identify serum or plasma molecular biomarkers to predict NDAF after IS or TIA, and evaluate their performance, utility and usability in clinical practice and research.

## Methods

A systematic literature review was undertaken using a pre-specified protocol in accordance with the Preferred Reporting Items for Systematic Reviews and Meta-Analyses (PRISMA-B) statement.^17^

### Data sources and searches

Searches were undertaken in MEDLINE (1946-28 December 2021), EMBASE (1947-28 December 2021), and Clinical Trials Registry using pre-defined search criteria and terms (Supplemental Tables 1 and 2). Hand searching of reference lists for additional eligible articles was conducted.

### Study selection

Published studies (English and non-English language) of hospitalised adults with IS, TIA or both, which reported use of any serum or plasma molecular biomarker and frequency of NDAF were included following screening of title and abstract by two reviewers (KW and AKK). Patients with known AF or PAF, or those with newly diagnosed AF on admission or within the first 24 h ECG were excluded. Studies reporting ECG monitoring for <24 h, participants without IS or TIA, or studies involving only haemorrhagic strokes were excluded. Corresponding authors were contacted by e-mail to resolve any issues relating to assessment of eligibility or data extraction. Discrepancies relating to eligibility or data extraction were resolved by discussion or arbitration to a third investigator (CJS).

### Data extraction

Data were independently extracted by two reviewers (KW and AKK) and included year of study and publication, study design, cohort size, country, cohort characteristics, name of molecular biomarker, assay methods, diagnostic criteria for NDAF, ECG monitoring method(s) for NDAF, duration of ECG monitoring, frequency of patients diagnosed with NDAF within each cohort, as well as discrimination (e.g. C-statistic) and calibration statistics.

### Objectives and outcome measures

The primary objective was identification of any molecular biomarker for predicting NDAF after IS/TIA. The primary outcome measure was any NDAF. Secondary analyses based on predefined subgroups were also performed, which included:

1. Use of biomarkers in unselected and selected patients, based on perceived risk using factors such as age and stroke pathogenesis (e.g. cryptogenic stroke).
2. The type and duration of ECG monitoring and NDAF.
3. The duration from qualifying IS/TIA to NDAF and relationship to molecular biomarkers.

### Assessment of quality: Risk of bias and applicability

Quality was assessed in terms of applicability and risk of bias, using the Quality Assessment of Diagnostic Accuracy (QUADAS)-2 tool,^18^ designed primarily for diagnostic studies. Diagnostic and prognostic studies share many common statistical features and QUADAS-2 allows the flexibility to select and tailor assessment of relevant domains. Judgement was made across four domains: patient selection; index biomarker, reference standard (extended ECG monitoring) and flow and timing. The QUADAS-2 tool was applied for each domain within the cohorts by two reviewers (KW and AKK) independently. Any discrepancy was resolved through discussion and where necessary, a third reviewer (CJS). Meta-analyses evaluating heterogeneity between studies were undertaken using StatsDirect (version3).

### Performance of molecular biomarkers

Performance measures including discrimination and calibration were described for each biomarker. For discriminative ability, we extracted information on the area under the receiver operating characteristic curve (AUC) or C-statistic and 95% confidence interval. We defined discriminative ability by c-statistic: >0.8, good; 0.6–0.8, modest and 0.5–0.6, poor.^19^

### Clinical usefulness

We described the applicability of biomarkers for initial screening after IS or TIA cohorts (unselected) and in selected higher-risk cohorts (e.g. ESUS or cryptogenic strokes). We incorporated categories of risk-stratification based on threshold levels (usability), and whether biomarkers were used to evaluate clinical management or clinician behaviours (utility). The generalisability of each biomarker was assessed by determining whether it was externally validated in an independent population.

## Results

### Search results

**Figure 1.**
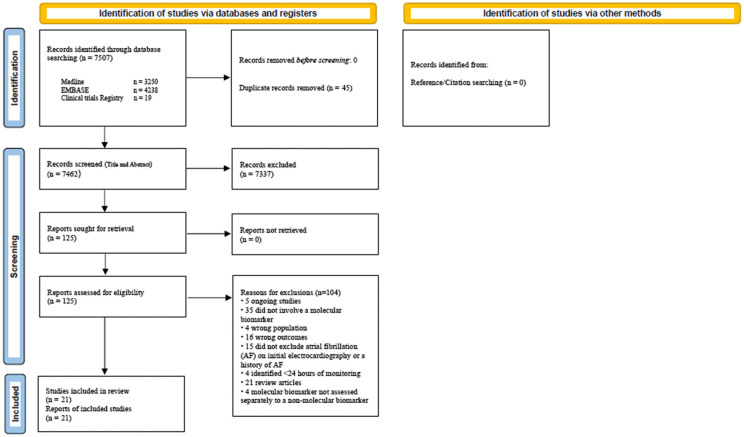
Study flow sheet.

The electronic search yielded 7449 publications. After screening, excluding duplicates and applying eligibility criteria, 123 full texts and abstracts were reviewed (Figure 1). Twenty-one published studies were eligible for inclusion.^20–40^

### Quality assessment

Overall, risk of bias was high (Supplemental Table 3). In some cohorts, risk of bias was high based on patient selection (exclusions based on incomplete data, NIHSS or age; selected higher-risk cohort; varying follow-up, and AF definition),^21,23,24,27–29,33,36,37,40^ index test application (use of biomarkers in selected patients only, thresholds derived from the same population tested),^37^ reference standard (NDAF detected through different extended ECG monitoring methods or applied inconsistently, lack of blinding to biomarker).^21,22^ Most studies were applicable to the study question.

### Study and patient characteristics

Characteristics of the 21 included studies are shown in Supplemental Table 4. Four thousand six hundred and forty participants were included (mean age 70 years, 42% female). Summary data for baseline vascular risk factors were incompletely reported for most studies (Supplemental Table 5). Stroke severity scale (NIHSS) was reported in 17 studies (81%) and median values ranged from 2 to 16. The median duration of monitoring overall was 4 days (range 1–548). A variety of cardiac monitoring approaches were utilised (Supplemental Figure 1 and Table 4). Definition of clinically significantly AF was variably reported, most commonly as >30 s in six studies (25%).^22,23,25,29,34,35^

### Molecular biomarkers for predicting NDAF

Twelve molecular biomarkers were identified (Supplemental Table 6). Most studies recorded cardiac biomarkers (17 studies) including natriuretic peptides (Brain natriuretic peptide, BNP; N-Terminal-pro Brain Natriuretic Peptide, NT-proBNP; N-Terminal pro-Atrial Natriuretic Peptide, NT-proANP; 13 studies)^22–25,28,29,31,32,34–36,38,40^ and cardiac troponins (Troponin-I, Troponin-T; four studies),^22,33,37,40^ involving majority (75%) of the included patients. BNP and NT-proBNP were evaluated in the majority of the participants (56%) with BNP evaluated in eight studies^29,31,32,34–36,38,39^ and NT-proBNP in six studies.^22–25,28,35^

Other biomarkers included haemostatic markers of prothrombotic state (d-dimer, Markers of Coagulation and Haemostatic Activation that is MOCHA Profile (serum d-dimer, prothrombin fragment 1.2, thrombin-antithrombin complex and fibrin monomer), Antithrombin III; six studies)^25–27,31,32,38^ markers of inflammation (high-sensitivity CRP; Erythrocyte sedimentation rate, ESR; three studies)^20,30,38^ and those that represent risk of cardiovascular disease (HbA1C, Creatinine) in eight studies.^21,22,25,31,32,35,38,39^

Over the last decade, there has been a trend towards evaluating cardiac and haemostatic biomarkers in higher risk selected IS patients including cryptogenic stroke patients (Supplemental Table 6). Among selected IS/TIA patients, seven studies evaluated cardiac biomarkers,^22–25,28,29,31^ most commonly NT-proBNP (five studies).^22–25,28^ Among haemostatic markers the MOCHA profile was most evaluated in four selected studies.^21,25,26,28^

### Performance and validation of the molecular biomarkers

Although variably reported, molecular biomarkers appeared to have modest to good discrimination (C-statistic 0.6–0.88). Discrimination metrics were only reported in two studies among non-cardiac biomarkers (Supplemental Table 2) and appeared to perform modestly (C-statistics 0.6–0.72)^24,26^; whereas reported cardiac biomarkers appeared to outperform non-cardiac biomarkers (C-statistics 0.66–0.88).^22,23,25,28,29,31–35,37–40^

Among selected cohorts (12 studies), the most common biomarkers were NT-proBNP (five studies; C-statistics reported by three studies, 0.69–0.83) and BNP (two studies; C statistics reported in two studies, 0.68–0.77; Supplemental Tables 6 and 7). Among unselected cohorts (nine studies), the most common biomarker was BNP (six studies; C-statistics reported in five studies, 0.75–0.88, Supplemental Tables 6 and 7). Calibration or goodness of fit measures were only determined in one unselected cohort when a concurrent risk score was derived.^32^ BNP was the only biomarker externally validated in two studies, although differing thresholds for NDAF were used (Supplemental Table 7)^31,32^ but at different sampling time points; in addition, one study didn’t specify assay method.^31^

### Clinical usefulness and utility of molecular biomarkers and implications in management

Clinical usefulness and utility can be best demonstrated through number to screen (NNS) values for extended ECG monitoring or recommendation for empirical oral anticoagulation; however, this was poorly reported. Only one small study recorded a baseline BNP cut off ⩾100 pg/ml, which reduced NNS with extended ECG monitoring from 18 to 3.^29^ No study evaluated empirical oral anticoagulation, although one study suggested a normal MOCHA profile on antiplatelet therapy was unlikely to benefit from early anticoagulation.^27^ Small sample sizes and lack of external validation limited generalisability.

## Discussion

In this systematic review, we identified 12 molecular biomarkers for NDAF prediction (detected with extended ECG monitoring) following IS or TIA (Supplemental Tables 6 and 7). There are a number of candidate biomarkers that could predict paroxysmal or occult AF including ECG measures, echocardiography and other imaging measures of the left atrium and molecular biomarkers.^41^ Of these, molecular biomarkers show most promise and would be easy to implement into clinical practice. Natriuretic peptides (BNP, NT-pro-BNP and Pro-ANP) were the most studied cardiac biomarkers (56% of study population) with modest to good discrimination. ANP and BNP are released from cardiac atria and ventricles, respectively, and have been shown to be associated with AF and cardioembolic strokes.^13,16,42^ However, several thresholds for these assays have been proposed which limit generalisability and utility. It is also important to consider that levels of natriuretic peptides can be influenced by heart failure, ischaemic heart disease and renal failure, and medications such as angiotensin converting enzyme inhibitors, limiting their utility in clinical practice.^43^ One other potential reason for reduced sensitivity of cardiac biomarkers in predicting PAF could also be that very brief runs of PAF do not produce the haemodynamic compromise required to cause detectable levels of these biomarkers.^34^ Further, stroke itself may also influence biomarkers, as post-stroke inflammation is associated with release of pro-inflammatory cytokines and inflammatory markers such as CRP and d-dimer. Another cardiac biomarker, Midregional pro-atrial natriuretic peptide (MR-proANP), which is a fragment of the prohormone to ANP was shown to be independently associated with NDAF in the CoRisk study^44^ and externally validated in a separate cohort (in the exact time frame since stroke symptom onset and using same assay methodology) within the BIOSIGNAL study. This is an interesting candidate molecular biomarker that has been shown to improve diagnostic accuracy in conjunction with several clinical risk scores, to predict NDAF after IS/TIA and needs further evaluation.^45^ Data from these studies were not included in the systematic review as the CoRisk study did not specify C-statistic values with extended ECG monitoring and the results from the BIOSIGNAL study were published after our search window.

An ideal biomarker would have a 100% sensitivity and specificity. For example, BNP measured in an unselected cohort with a threshold 131 pg/ml^33^ demonstrated 98% sensitivity, suggesting that only 2% of NDAF would not be predicted; although with 71% specificity which means ~29% who did not have NDAF were incorrectly identified as higher risk (false positive). Similarly, in a small study involving cryptogenic stroke patients, NT-proBNP <505 pg/ml (within 24 h of stroke admission) had a negative predictive value of 98% suggesting a potential role in identifying those who may least benefit from extended cardiac monitoring.^24^ These statistics are very useful, particularly if considering inexpensive tests like 12 lead ECG or pulse checks for screening purposes and a biomarker with high sensitivity. Indeed, biomarkers may be better placed to ‘rule out’ (rather than rule in) when selecting people for extended cardiac monitoring.^46^ However, if more expensive monitoring techniques like ILRs are considered for higher risk people then high false positive rates may be an issue.

Molecular biomarkers can also be used in conjunction with clinical demographics to develop risk scores.^47^ For example, iPAB score^32^ (history of arrhythmia or antiarrhythmic agent use, atrial dilation and BNP elevation) of ⩾4 had 95% specificity, suggesting possible targetted use of expensive monitoring devices. This approach is more relevant now considering societal recommendations that highlight the importance of more prolonged cardiac monitoring to search for AF after IS or TIA.^48^ Indeed, a composite model with multiple molecular biomarkers or which include clinical parameters and/or ECG, echocardiography and molecular biomarkers could improve predictive ability and should be prospectively evaluated.^41^

It was not possible to determine the temporal profile of molecular biomarkers in relation to NDAF. The half-life of cardiac biomarkers varies substantially. NT-proBNP and NT-proANP have a longer half-life (122 and 60–120 min respectively) than BNP or ANP (22 and 2 min respectively) and are more readily detectable.^49^ This may have clinical and research implications as not all stroke patients present early. In this study, all cardiac biomarkers appeared to have been analysed within 72 h of hospital admission. Only two studies^28,34^ evaluated cardiac biomarkers at serial intervals and median BNP/NT-proBNP levels were higher at all time points for patients with NDAF. One study evaluated MOCHA profile at serial intervals (2–4 weeks, then at 6 weeks) from stroke and higher levels corresponded with NDAF.^26^

The lack of standardised assays for some biomarkers and lack of studies evaluating cost effectiveness and recurrent stroke risk are potentially limiting factors in clinical practice. Of note, this review identified several immunoassay methods when evaluating molecular biomarkers (Supplemental Table 8). Some cardiac biomarkers are associated with atrial cardiopathy,^50,51^ a condition which suggests atrial structural and functional disorder that could precede AF and where empirical anticoagulation could potentially be beneficial even in the absence of proven AF. Coagulation markers such as the MOCHA profile are also thought to be associated with atrial cardiopathy.^24,26^ Several randomised studies evaluating these markers of atrial cardiopathy are currently in progress.^52–54^

Our findings were limited by small sample sizes and incomplete reporting. Patient selection and eligibility varied significantly. The definition of PAF deemed clinically significant also varied and was not reported by many studies (Supplemental Table 4). The duration of cardiac monitoring prior to inclusion varied substantially and we found that NDAF frequency was highly variable, with higher detection rates tending to be associated with more prolonged monitoring and in cryptogenic stroke or ESUS patients (Supplemental Figure 1), in keeping with a previous systematic review.^10^ NDAF among unselected patients was 11.8% (6.8%–16.8%, I^2^ = 81.1) and among selected higher risk patients was 14.9% (8.2%–21.6%, I^2^ = 88.1). NDAF frequency was highly variable even among high-risk cryptogenic stroke studies possibly due to the absence of standardised investigations to diagnose cryptogenic stroke. Follow-up periods were different among studies also contributing to heterogeneity (Supplemental Table 4). There was substantial selection bias (46%; Supplemental Table 3) with molecular biomarkers sometimes requested at clinician discretion in retrospective studies and exclusion of potentially eligible patients who were not admitted to the stroke unit.

## Conclusion

Molecular biomarkers, especially cardiac biomarkers, may help to stratify risk of NDAF after IS or TIA and should be further evaluated in prospective studies with rigorous follow-up. Larger studies are needed to determine cost effectiveness, a standardised definition of clinically significant AF, patient investigation pathways and optimal approaches for extended ECG monitoring.

## Supplemental Material

sj-docx-1-eso-10.1177_23969873221136927 – Supplemental material for Molecular biomarkers predicting newly detected atrial fibrillation after ischaemic stroke or TIA: A systematic review Click here for additional data file. Supplemental material, sj-docx-1-eso-10.1177_23969873221136927 for Molecular biomarkers predicting newly detected atrial fibrillation after ischaemic stroke or TIA: A systematic review by Kirsty Ward, Andy Vail, Alan Cameron, Mira Katan, Gregory YH Lip, Jesse Dawson, Craig J Smith and Amit K Kishore in European Stroke Journal

sj-docx-2-eso-10.1177_23969873221136927 – Supplemental material for Molecular biomarkers predicting newly detected atrial fibrillation after ischaemic stroke or TIA: A systematic review Click here for additional data file. Supplemental material, sj-docx-2-eso-10.1177_23969873221136927 for Molecular biomarkers predicting newly detected atrial fibrillation after ischaemic stroke or TIA: A systematic review by Kirsty Ward, Andy Vail, Alan Cameron, Mira Katan, Gregory YH Lip, Jesse Dawson, Craig J Smith and Amit K Kishore in European Stroke Journal
