# Supplementary material for: Molecular biomarkers predicting newly detected atrial fibrillation after ischaemic stroke or TIA: A systematic review
Source: Eur Stroke J. 2022 Dec 6;8(1):125–31. doi: 10.1177/23969873221136927 (PMC10069198; doi:10.1177/23969873221136927)
Supplement: sj-docx-1-eso-10.1177_23969873221136927 – Supplemental material for Molecular biomarkers predicting newly detected atrial fibrillation after ischaemic stroke or TIA: A systematic review [file sj-docx-1-eso-10.1177_23969873221136927.docx]

**Online Only Supplement**

**Molecular biomarkers predicting newly detected atrial fibrillation after ischaemic stroke or TIA: A systematic review**

Kirsty Ward ^1^, Andy Vail ^2^, Alan Cameron ^3^, Mira Katan^4^, Gregory Y. H. Lip ^5,6^, Jesse Dawson ^3^, Craig J. Smith ^1,7^, Amit K. Kishore ^1,7^

^1^Manchester Center for Clinical Neurosciences, Geoffrey Jefferson Brain Research Centre, Manchester Academic Health Science Centre, Salford Care organisation, Northern Care Alliance NHS Foundation Trust, UK; ^2^Centre for Biostatistics, University of Manchester, Manchester Academic Health Science Centre, UK; ^3^Institute of Cardiovascular and Medical Sciences, University of Glasgow; ^4^Stroke Center/ Dept. Of Neurology University Hospital of Basel, Switzerland; ^5^Liverpool Centre for Cardiovascular Sciences at University of Liverpool, Liverpool John Moores University and Liverpool Heart & Chest Hospital, Liverpool, United Kingdom; ^6^Department of Clinical Medicine, Aalborg University, Aalborg, Denmark; ^7^ Division of Cardiovascular Sciences, Faculty of Biology, Medicine and Health, University of Manchester, Manchester, UK

**Online Only Supplement Table 1: Eligibility Criteria**

**Inclusion Criteria**

1. English or non-English language articles
2. Adult age participants (≥ 18 years)
3. Prospective, retrospective observational studies, quasi-experimental or randomised control trials.
4. Studies were included from 1946 – 28 December 2021
5. Studies must have included molecular biomarkers that predict any newly detected atrial fibrillation after ischaemic stroke/transient ischaemic attack (TIA).
6. Studies must have included the reporting of any newly detected AF following ischaemic stroke or TIA
7. Studies must have used extended Electrocardiography (ECG) monitoring (≥24h) for reporting newly detected atrial fibrillation (AF) following ischaemic stroke or TIA
8. All sub-categories of AF were included

**Exclusion Criteria**

1. Studies involving participants with only haemorrhagic stroke were excluded
2. Participants with a known or determined history of AF preceding their acute ischaemic stroke or TIA were excluded.
3. Studies not using extended ECG monitoring to determine new AF were excluded.
4. Studies published only in abstract form were excluded from inclusion into qualitative synthesis

**Online Only Supplement Table 2: Search strategy**

| Search Areas | Thesaurus terms | Free text terms |
| --- | --- | --- |
| EMBASE | Subject Search in EMTREE: exp *Cerebrovascular disease/  exp *Biological marker/  exp *Atrial fibrillation/ | Stroke*, “CVA”, “cerebrovascular accident”, “TIA”, “transient ischaemic attack”, “transient ischaemic attack”, marker*, biomarker*, “AF”, “atrial fibrillation” |
| Medline | Subject Search in MeSH:  exp *Cerebrovascular disorders/  exp *Biomarkers/  exp *Atrial fibrillation/ | Stroke*, “CVA”, “cerebrovascular accident”, “TIA”, “transient ischaemic attack”, “transient ischaemic attack”, marker*, biomarker*, “AF”, “atrial fibrillation |
| Clinical Trials Registry |  | Ischaemic stroke, atrial fibrillation, biomarkers |

**Online Only Supplement Table 3:** **QUADAS-2 quality assessment of included studies**

| **Authors** | **Risk of bias** | | | Flow/timing | **Applicability** | | |
| --- | --- | --- | --- | --- | --- | --- | --- |
|  | Patient selection | Index test | Reference standard |  | Patient selection | Index test | Reference standard |
| Naya et al, 2008 | High | Low | Unclear | Low | Low | Low | Low |
| Bugnicourt et al ,2010 | High | Low | Low | Low | Low | Low | Low |
| Okada et al, 2010 | Unclear | Unclear | Unclear | Low | Low | Low | Unclear |
| Shibazaki et al, 2012 | Low | Unclear | Unclear | Unclear | Low | Low | Low |
| Wachter et al, 2012 | low | Low | Low | Low | Low | Low | Low |
| Beaulieu-Boire et al, 2013 | High | Unclear | Unclear | Low | Low | Unclear | Low |
| Rodriguez-Yanez et al, 2013 | High | Low | Unclear | High | Low | Low | High |
| Suissa et al, 2013 | Low | Unclear | Low | Low | Low | Low | Low |
| Fonseca et al, 2014 | High | Unclear | Low | Low | Low | Low | Low |
| Sanak et al, 2015 | Low | Low | High | Low | Low | Low | High |
| Ward et al, 2015 | High | High | Unclear | Unclear | Low | Unclear | Unclear |
| Yoshioka et al, 2015 | Low | Unclear | Low | Low | Low | Low | Low |
| Carrazco et al, 2018 | High | Low | High | Unclear | Low | Low | Low |
| Ellis et al, 2018 | High | Low | Low | Low | Low | Low | Low |
| Hawkes et al, 2018 | Unclear | Unclear | Unclear | Unclear | Low | Low | Unclear |
| Acampa et al,2019 | Low | Unclear | Low | Low | Low | Low | Low |
| Kneihsl et al, 2019 | Low | Low | Low | Low | Low | Low | Low |
| Miyazaki et al, 2020 | Low | Unclear | Unclear | Low | Low | Low | Low |
| Nahab et al, 2020 | Low | Low | Low | Low | Low | Low | Low |
| Pagola et al, 2020 | High | Unclear | Low | Unclear | Low | Low | Low |
| Wasser et al, 2020 | High | Low | Low | Low | Low | Low | Low |

QUADAS-2= Quality Assessment of Diagnostic Accuracy (QUADAS)-2 tool

**Online Only Supplement Table 4: Study characteristics**

| ***Author*** | ***Year Published*** | ***Country*** | ***Study period*** | ***Study type*** | ***Diagnosis*** | ***AF duration*** | ***Time interval to initial monitoring (median,d)*** | ***ECG monitoring*** | ***Study end point/Follow-up (mean)*** |
| --- | --- | --- | --- | --- | --- | --- | --- | --- | --- |
| **Naya** et al | 2008 | Japan | 2005-2006 | Prospective | IS | Any AF | NR | 24h Holter+14d | NR |
| **Bugnicourt** et al | 2010 | France | 2006-2007 | Retrospective | IS/TIA | Any AF | NR | 48h Holter | NR |
| **Okada** et al | 2010 | Japan | 2006-2007 | Prospective | IS/TIA | Any AF | <7d | IP telemetry+24h Holter | NR (IP only) |
| **Shibazaki et al** | 2012 | Japan | 2006-2010 | Prospective | IS | Any AF | <24h | IP telemetry+24h Holter | NR (IP only) |
| **Beaulieu-Boire et al** | 2012 | Canada | 2006-2010 | Prospective | IS/TIA | Any AF | NR | 24h Holter | 3m |
| **Wachter** et al | 2012 | Germany | 2009-2010 | Prospective | IS/TIA | ≥30s | <24h | 7d Holter | NR |
| **Rodriguez-Yanez** et al | 2013 | Spain | 2008-2011 | Prospective | IS | Any AF | <72h | IP telemetry±24h ECG | 18m |
| **Suissa et al** | 2013 | France | 2010-2011 | Prospective | IS | ≥30s | <24h | IP telemetry or Holter | NR (IP only) |
| **Fonseca** et al | 2014 | Portugal | 2009-2010 | Prospective | IS/TIA | ≥30s | <7d | Serial 24h Holter | At least 6m |
| **Sanak** et al | 2014 | Czech Republic | 2011-2014 | Retrospective | IS | ≥30s | 42d | 21d | NR |
| **Ward** et al | 2015 | Ireland | 2012-2013 | Retrospective | IS/TIA | Any AF | NR | 24h IP telemetry or Holter | NR |
| **Yoshioka et al** | 2015 | Japan | 2013-2014 | Prospective | IS | Any AF | <24h | IP telemetry+24h Holter | NR (IP only) |
| **Hawkes** et al | 2018 | Argentina | 2012-2013 | Retrospective | IS | Any AF | NR | NR(non-invasive only) | 12m |
| **Ellis** et al | 2018 | USA | 2015-2016 | Retrospective | IS | Any AF | NR | 30d MCOT/ILR | 16m |
| **Carrazco** et al | 2018 | USA | 2013-2015 | Retrospective | IS | ≥2min | 4.2d | ILR | 8m |
| **Kneihsl** et al | 2019 | Austria | 2017-2018 | Prospective | IS | ≥30s | <24h | 48h IP telemetry +24h Holter | 12m |
| **Wasser etal** | 2019 | Germany | 2013-2014 | Randomized | IS | ≥30s | NR | 10d Holter (0,3m,6m) | NR |
| **Acampa et al** | 2019 | Italy | NR | Prospective | IS | >5min | NR | 7d | NR |
| **Miyazaki et al** | 2020 | Japan | 2012-2017 | Retrospective | IS | Any AF | <7d | IP telemetry+24h Holter | NR |
| **Pagola et al** | 2020 | Spain | 2015-2017 | Prospective | IS | PAF>5h | <72h | 28d Holter | 24m |
| **Nahab** et al | 2020 | USA | 2017-2018 | Retrospective | IS | Any AF | NR | MCOT/ILR | 10m |

IS=Ischaemic Stroke, TIA=Transient Ischaemic Attack, NR=Not Reported, PAF= Paroxysmal Atrial Fibrillation., IP=inpatient, ECG=electrocardiography, MCOT=mobile cardiac outpatient telemetry, ILR=Implantable loop recorder

**Online Only Supplement Table 5: Patient characteristics**

| ***Author*** | ***Age (Mean, y)*** | ***Male (%)*** | ***NIHSS (median value)*** | ***DM (%)*** | ***HTN (%)*** | ***Obesity (%)*** | ***Prior stroke (%)*** | ***IHD (%)*** |
| --- | --- | --- | --- | --- | --- | --- | --- | --- |
| Naya et al, 2008 | 70 | 67 | 13* | 30 | 64 | NR | NR | NR |
| Bugnicourt et al, 2010 | 65 | 58 | NR | 20.6 | 58.7 | NR | 15.4 | 10.7 |
| Okada et al, 2010 | 70 | 68 | 5 | 27 | 55 | NR | 23.6 | NR |
| Shibazaki et al, 2012 | 72.4* | 64 | 4.6* | 25.6 | 65.9 | NR | 20 | 10 |
| Beaulieu-Boire et al | 70.4* | 52 | 3 | 26.7 | 68.6 | NR | 22.3 | 27.4 |
| Wachter et al, 2012 | 68.1* | 58.2 | 4* | 22.7 | 72.7 | NR | 15 | 15 |
| Rodriguez-Yanez et al, 2013 | 70.6 | 57 | 7 | 25.7 | 52 | NR | 9.4 | NR |
| Suissa et al, 2013 | 62.5 | 63 | 7 | 9.7 | 45 | NR | 8.3 | 10.7 |
| Fonseca et al, 2014 | 63.8 | 57 | 7* | 26 | 58.1 | NR | 27.7 | 16.3 |
| Sanak et al, 2014 | 39 | 49 | 9 | 1 | 8 | NR | 1.5 | 0 |
| Ward et al | 73 | 53 | NR | NR | NR | NR | NR | NR |
| Yoshioka et al, 2015 | 74.4* | 61 | NR | 29.8 | 77.4 | NR | 13.8 | 13.2 |
| Hawkes et al, 2018 | 68 | NR | 2 | NR | 61 | NR | 15 | NR |
| Ellis et at, 2018 | 60 | 38 | NR | 37 | 71 | 0 | 9.5 | 7.1 |
| Carrazco et al, 2018 | 66 | 47 | 5 | 27 | 78 | 30 | 15 | NR |
| Kneihsl et al, 2019 | 67.9* | 57 | 4* | 15.1 | 80.8 | NR | NR | NR |
| Wasser et al 2019 | 72.6* | 59 | 3 | 28.2 | 79.9 | NR | 20.1 | 15.3 |
| Acampa et al 2019 | 73,1* | 49 | NR | 14 | 55 | NR | 2 | 14 |
| Miyazaki et al, 2020 | 76 | 58 | 4 | 22 | 74 | NR | 17 | 13 |
| Pagola et al, 2020 | 75 | 50 | 4 | 26.2 | 76.7 | NR | NR | NR |
| Nahab et al, 2020 | 64 | 39 | NR | 21 | 79 | NR | 9.8 | NR |

HTN=Hypertension, IHD=Ischaemic Heart Disease, DM=diabetes mellitus, CCF= Congestive Cardiac Failure, NIHSS=National Institutes For Health Stroke Scale, NR=Not Reported.*weighted average

**Online Only Supplement Table 6: Molecular Biomarkers**

| **Author** | **Size(n)** | **Cardiac biomarkers** | | | | |  | **Non-cardiac biomarkers** | | | | | | | |
| --- | --- | --- | --- | --- | --- | --- | --- | --- | --- | --- | --- | --- | --- | --- | --- |
|  |  | **BNP** | **NT-ProBNP** | **Troponin I** | **Troponin T** | **NT-Pro ANP** |  | **D-Dimer** | **MOCHA Profile** | **Antithrombin III** | **CRP** | **ESR** | **Hba1c** | **Creatinine** |  |
| **Unselected Studies (by publication year)** | | | | | | | | | | | | | | |  |
| **Naya et al, 2008** | 53 | **✓** |  |  |  |  |  |  |  |  |  |  |  |  |  |
| **Bugnicourt, 2010** | 402 |  |  | **✓** |  |  |  |  |  |  |  |  |  |  |  |
| **Okada et al, 2010** | 165 | **✓** |  |  |  |  |  | **✓** |  |  | **✓** |  |  | **✓** |  |
| **Shibazaki et al, 2012** | 584 | **✓** |  |  |  |  |  |  |  |  |  |  |  | **✓** |  |
| **Beaulieu-Boire et al, 2012** | 278 |  |  | **✓** |  |  |  |  |  |  |  |  |  |  |  |
| **Wachter et al, 2012** | 220 | **✓** | **✓** |  |  | **✓** |  |  |  |  |  |  |  | **✓** |  |
| **Suissa et al, 2013** | 300 | **✓** |  |  |  |  |  |  |  |  |  |  |  |  |  |
| **Yoshioka et al, 2015** | 143 | **✓** |  |  |  |  |  | **✓** |  |  |  |  |  | **✓** |  |
| **Ward et al, 2015** | 133 |  |  | **✓** |  |  |  |  |  |  |  |  |  |  |  |
| **Selected Studies (by publication year)** | | | | | | | | | | | | | | |  |
| **Rodriguez-Yanez et al, 2013** | 264 |  | **✓** |  |  |  |  |  |  |  |  |  |  |  |  |
| **Fonseca et al, 2014** | 80 |  | **✓** |  |  |  |  |  |  |  |  |  |  |  |  |
| **Sanak et al, 2015** | 95 |  | **✓** |  | **✓** |  |  |  |  |  |  |  | **✓** | **✓** |  |
| **Hawkes et al, 2018** | 77 |  |  |  |  |  |  |  |  |  |  | **✓** |  |  |  |
| **Ellis et al, 2018** | 42 |  |  |  |  |  |  |  | **✓** |  |  |  |  |  |  |
| **Carrazco et al, 2018** | 100 |  |  |  |  |  |  |  |  |  |  |  | **✓** |  |  |
| **Kneihsl et al, 2019** | 143 |  | **✓** |  |  |  |  | **✓** |  | **✓** |  |  |  | **✓** |  |
| **Wasser et al, 2020** | 187 | **✓** |  |  |  |  |  |  |  |  |  |  |  |  |  |
| **Acampa et al, 2019** | 222 |  |  |  |  |  |  |  |  |  | **✓** |  |  |  |  |
| **Miyakazi et al, 2020** | 206 | **✓** |  |  |  |  |  | **✓** |  |  |  |  |  | **✓** |  |
| **Pagola et al, 2020** | 253 |  | **✓** |  |  |  |  |  |  |  |  |  |  |  |  |
| **Nahab et al, 2020** | 132 |  |  |  |  |  |  |  | **✓** |  |  |  |  |  |  |

BNP=Brain Natriuretic Peptide; NT-Pro BNP=N-Terminal Brain Natriuretic Peptide; NT Pro ANP=N-Terminal Pro Atrial Natriuretic Peptide; CRP=C-reactive protein; ESR=Erythrocyte Sedimentation rate

**Online Only Supplement Table 7: Performance of Molecular Biomarkers**

| Biomarker | Patient selection | Author | Event to index test | AUC (C-statistic, 95% CI) |  |
| --- | --- | --- | --- | --- | --- |
| Cardiac | | |  |  |  |
| BNP | Unselected IS | Naya et al, 2008 | <12h | NR |  |
|  |  | Shibazaki et al, 2012 | <12h | 0.82 (NR) |  |
|  |  | Suissa et al, 2013 | <12h | 0.87 (NR) |  |
|  |  | Yoshioka et al, 2015 | <7d | 0.88 (NR) |  |
|  | Unselected IS and TIA | Okada et al, 2010 | <12h | 0.83 (NR) |  |
|  |  | Wachter et al, 2012 | 0h, 6h, 24h | 0.75 (0.66-0.83) |  |
|  | Selected ESUS | Miyakazi et al, 2020 | 0h | 0.77 (NR) |  |
|  | Selected IS >60y | Wasser et al, 2020 | 72h and 3m | 0.71 (0.6-0.82) |  |
|  |  |  |  |  |  |
| NT-pro BNP | Unselected IS and TIA | Wachter et al, 2012 | 0h, 6h and 24h | 0.64 (0.53-0.75) |  |
|  | Cryptogenic IS | Rodriguez-Yanez et al, 2013 | <24h | NR |  |
|  |  | Fonseca et al, 2014 | <72h | 0.83 (0.73-0.92) |  |
|  |  | Kneihsl et al, 2019 | NR | 0.88 (0.84-0.93) |  |
|  | Selected cryptogenic IS <50y | Sanak et al, 2015 | <12h | NR |  |
|  | Selected cryptogenic IS >55y | Pagola et al, 2020 | <72h | 0.69 (NR) |  |
| NT-proANP | Unselected IS and TIA | Wachter et al, 2012 | 0h, 6h,24h | 0.66 (0.56-0.76) |  |
| Troponin I | Unselected IS and TIA | Bugnicourt et al, 2010 | <24h | 0.66 (0.53-0.79) |  |
|  |  | Beaulieu-Boire et al, 2013 | <12h | NR |  |
|  |  | Ward et al, 2015 | <24h | 0.71 (NR) |  |
| Troponin T | Selected cryptogenic IS <50y | Sanak et al, 2015 | <12h | 0.66 (NR) |  |
| Non-cardiac | | | | | |
| D-dimer (μg/ml) | Unselected IS | Yoshioka et al, 2015 | 24h | NR |  |
|  | Unselected IS and TIA | Okada et al, 2010 | <12h | NR |  |
|  | Selected ESUS | Kneihsl et al, 2019 | <24h | 0.6 (0.52-0.67) |  |
|  |  | Miyazaki et al, 2020 | <24h | NR |  |
| MOCHA profile | Selected ESUS | Ellis et al, 2018 | >2w | 0.72 (NR) |  |
|  |  | Nahab et al, 2020 | >2w | NR |  |
| Antithrombin III (%) | Selected ESUS | Kneihsl et al, 2019 | <24h | 0.64 (0.57-0.71) |  |
| hs-CRP (mg/dL) | Unselected IS and TIA | Okada et al, 2010 | <12h | NR |  |
|  | Selected cryptogenic | Acampa et al, 2019 | NR | NR |  |
| ESR (mm/h) | Selected ESUS | Hawkes et al, 2018 | NR | NR |  |
| HbA1C (mmol/L) | Selected cryptogenic ≤50 years | Sanak et al, 2015 | <60h | NR |  |
|  | Selected ESUS | Carrazco et al, 2018 | NR | NR |  |
| Creatinine (mg/dL) | Unselected IS | Shibazaki et al, 2012 | NR | NR |  |
|  |  | Yoshioka et al, 2015 | NR | NR |  |
|  | Unselected IS and TIA | Okada et al, 2010 | NR | NR |  |
|  |  | Wachter et al, 2012 | NR | NR |  |
|  | Selected ESUS | Kneihsl et al, 2019 | <24h | NR |  |
|  |  | Miyazaki et al, 2020 | NR | NR |  |
|  | Selected cryptogenic ≤50 years | Sanak et al, 2015 | NR | NR |  |
| BNP= Brain Natriuretic Peptide; NT=Pro BNP=N Terminal Brain Natriuretic Peptide; NT Pro-ANP=N Terminal pro Atrial Natriuretic Peptide;  CRP=high-sensitivity C-reactive protein, ESR=Erythrocyte sedimentation rate; ESUS=Embolic stroke of Unknown source; IS=Ischaemic stroke;  MOCHA=serum D-dimer, prothrombin fragment 1.2, thrombin -antithrombin complex, and fibrin monomer; PAF=paroxysmal AF; TIA=Transient ischaemic attack | | | | | |

**Online Only Supplement Table 8: Assay Methods**

| **Author** | **Biomarker** | **Assay** |
| --- | --- | --- |
| Acampa et al, 2019 | CRP | Particle-enhanced turbidimetric method using module C501 on a Cobas-6000 platform (Roche, Switzerland) |
| Beaulieu-Boire et al, 2013 | Troponin I | NR |
| Bugnicourt et al, 2010 | Troponin I | LOCI immunoassay (Dade Behring, USA) |
| Carrazco et al, 2018 | HbA1C | NR |
| Ellis et al, 2018 | MOCHA profile | 3.2 citrated plasma, D dimer performed via high sensitivity latex dimer assay (Instrumentation Laboratories, US), prothrombin fragment and thrombin-antithrombin performed via Enzygnost ELISA kit (Siemens Healthcare, US) fibrin monomer by latex immunoassay (Stago, USA) |
| Fonseca et al, 2014 | NT-proBNP | Electrochemiluminescence using the Elecsys 2010 immunoassay analyser (Roche, Switzerland) |
| Hawkes et al, 2018 | ESR | NR |
| Kneihsl et al, 2019 | NT-proBNP | Electrochemiluminescence using the Cobas 8000 immunoassay analyser (Roche Diagnostics, Germany) |
|  | D dimer | Immunoturbidimetric/photometric method using the Atellica COAG 360 system (Siemens Healthineers, Germany) |
|  | Antithrombin III | Immunoturbidimetric/photometric method using the Atellica COAG 360 system (Siemens Healthineers, Germany) |
|  | Creatinine | NR |
| Miyazaki et al, 2020 | BNP | NR |
|  | D dimer | NR |
|  | Creatinine | NR |
| Nahab et al, 2020 | MOCHA profile | 3.2 citrated plasma, D dimer performed via high sensitivity latex dimer assay (Instrumentation Laboratories, US), prothrombin fragment and thrombin-antithrombin performed via Enzygnost ELISA kit (Siemens Healthcare, US) fibrin monomer by latex immunoassay (Stago, USA) |
| Naya et al, 2008 | BNP | Specific sandwich immunoradiometric assay using Shionoria BNP kit (Shionogi, Japan) |
| Okada et al, 2010 | BNP | Specific sandwich immunoradiometric assay using Shionoria BNP kit (Shionogi, Japan) |
|  | D dimer | NR |
|  | CRP | NR |
|  | Creatinine | NR |
| Pagola et al, 2020 | NT-ProBNP | Electrochemiluminescence immunoassay using the Cobas 8000 analyser (Roche Diagnostics, Germany) |
| Rodriguez-Yanez et al, 2013 | NT-proBNP | Electrochemiluminescence immunoassay using the Elecsys 2010 immunoassay analyser (Roche, Switzerland) |
| Sanak et al, 2015 | NT-proBNP | NR |
|  | Troponin T | NR |
|  | HbA1C | NR |
|  | Creatinine | NR |
| Shibazaki et al, 2012 | BNP | Specific sandwich immunoradiometric assay using Shionoria BNP kit (Shionogi, Japan) |
|  | Creatinine | NR |
| Suissa et al, 2013 | BNP | Sandwich chemiluminescence immunoassay using the Alere Triage BNP test with an Access 2 immunoassay system (Beckman-Coulter, USA) |
| Wachter et al, 2012 | BNP | Sandwich chemiluminescence immunoassay using Centaur (Bayer Vital, Germany) |
|  | NT-proBNP | Sandwich enzyme immunoassay (Roche, Switzerland) |
|  | NT-proANP | ELISA (Biomedica, Austria) |
|  | Creatinine | NR |
| Ward et al, 2015 | Troponin I | Chemiluminescent immunoassay AccuTnI+3 (Beckman Coulter Inc, USA) |
| Wasser et al, 2020 | BNP | Sandwich chemiluminescence immunoassay on the ADVIA Centaur CP Immunoassay System (Siemens Healthineers, Germany) |
| Yoshioka et al, 2015 | BNP | Specific sandwich immunoradiometric assay using Shionoria BNP kit (Shionogi, Japan) |
|  | D dimer | NR |
|  | Creatinine | NR |

BNP: brain natriuretic peptide; CRP: C-reactive protein; EDTA: ethylenediaminetetraacetic acid; ELISA: Enzyme-Linked Immunosorbent Assay; ESR: erythrocyte sedimentation rate; LOCI: Luminescent Oxygen Channeling Immunoassay; MOCHA: markers of coagulation and haemostatic activation; NR: not reported; NT-proANP: N-terminal pro-atrial natriuretic peptide NT-proBNP: N-terminal pro-brain natriuretic peptide.
