# Supplementary figures and images for: Molecular biomarkers predicting newly detected atrial fibrillation after ischaemic stroke or TIA: A systematic review
Source: Eur Stroke J. 2022 Dec 6;8(1):125–31. doi: 10.1177/23969873221136927 (PMC10069198; doi:10.1177/23969873221136927)

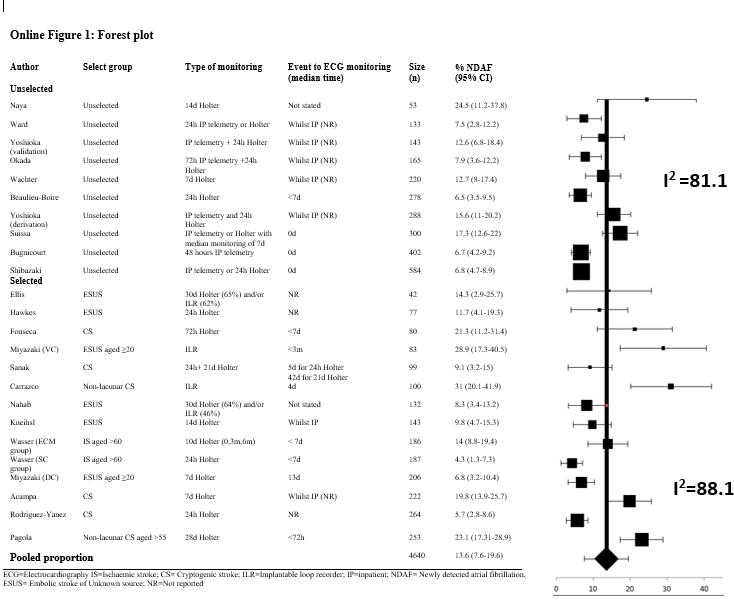

Supplement: sj-docx-2-eso-10.1177_23969873221136927 – Supplemental material for Molecular biomarkers predicting newly detected atrial fibrillation after ischaemic stroke or TIA: A systematic review [file sj-docx-2-eso-10.1177_23969873221136927.PNG]
